# Low‐level laser and adipose‐derived stem cells altered remodelling genes expression and improved collagen reorganization during tendon repair

**DOI:** 10.1111/cpr.12580

**Published:** 2019-02-07

**Authors:** Letícia D. Lucke, Fernanda O. Bortolazzo, Viviane Theodoro, Lucas Fujii, André L. Bombeiro, Maíra Felonato, Rodrigo A. Dalia, Giane D. Carneiro, Luciana P. Cartarozzi, Cristina Pontes Vicente, Alexandre L. R. Oliveira, Fernanda A. S. Mendonça, Marcelo A. M. Esquisatto, Edson R. Pimentel, Andrea A. de Aro

**Affiliations:** ^1^ Department of Structural and Functional Biology, Institute of Biology University of Campinas – UNICAMP Campinas São Paulo Brazil; ^2^ Biomedical Sciences Graduate Program Herminio Ometto University Center ‐ UNIARARAS Araras São Paulo Brazil

**Keywords:** cell therapy, decorin, GDF‐5, healing, photobiostimulation, tenomodulin

## Abstract

**Objectives:**

The cellular therapy using adipose‐derived mesenchymal stem cells (ASCs) aims to improve tendon healing, considering that repaired tendons often result in a less resistant tissue. Our objective was to evaluate the effects of the ASCs combination with a low‐level laser (LLL), an effective photobiostimulation for the healing processes.

**Materials and methods:**

Rats calcaneal tendons were divided into five groups: normal (NT), transected (T), transected and ASCs (SC) or LLL (L), or with ASCs and LLL (SCL).

**Results:**

All treated groups presented higher expression of *Dcn* and greater organization of collagen fibres. In comparison with T, LLL also up‐regulated *Gdf5 *gene expression, ASCs up‐regulated the expression of *Tnmd*, and the association of LLL and ASCs down‐regulated the expression of *Scx*. No differences were observed for the expression of *Il1b*, *Timp2*, *Tgfb1*, *Lox*, *Mmp2*, *Mmp8 and Mmp9*, neither in the quantification of hydroxyproline, TNF‐α, PCNA and in the protein level of Tnmd. A higher amount of IL‐10 was detected in SC, L and SCL compared to T, and higher amount of collagen I and III was observed in SC compared to SCL.

**Conclusions:**

Transplanted ASCs migrated to the transected region, and all treatments altered the remodelling genes expression. The LLL was the most effective in the collagen reorganization, followed by its combination with ASCs. Further investigations are needed to elucidate the molecular mechanisms involved in the LLL and ASCs combination during initial phases of tendon repair.

## 1 INTRODUCTION

The calcaneus tendon is one of the most affected tendons by ruptures, and its recovery after an injury turns to be a challenge in the current clinical practice.^1^ The calcaneus tendon may present reduced mechanical resistance in the elderly, after prolonged immobilization and after different types of injuries arising from tendinopathies, sports practices and daily activities, due to changes in the organization and composition of its extracellular matrix (ECM).^2^ During tendon repair, the formation of fibrous tissue occurs, less organized and with reduced biomechanical resistance, leading to the loss of part of the functions compared to the native tissue.^3^

In certain regions of the tendons, especially where there is accumulation of specific growth factors, there are mesenchymal stem cells (MSCs) native to the tissue. These cells decrease with age,^4^ and factors such as obesity, diabetes and hormonal changes also influence their proliferative and differentiation capacity.^5^ There is a growing search for methodologies that aim to stimulate the proliferation and differentiation of native tendon MSCs during the repair process.^5^, ^6^ Researches about the treatment of tendon injuries using adipose‐derived mesenchymal stem cells (ASCs) show a better organization of collagen bundles, as well as improved biomechanics and gait on treated animals.^6^

The photobiostimulation promoted by the low‐level laser (LLL) has attracted the attention of researchers in recent years, already being used in clinical practice for the treatment of tendinous and post‐operative injuries, accelerating the repair process, with the improvement of the tissue reorganization.^7^ Low‐level laser therapy promotes tissue repair mainly due to its anti‐inflammatory effect, but also has a mitogenic potential.^8^ Thus, the combination of LLL and ASCs in vivo could induce a greater proliferation of these cells in the injured region, considering small the percentage of ASCs that survive at the injury site after application.^9^

Although some in vitro studies demonstrate the efficiency of LLL treatment combined with MSCs,^8^, ^10^, ^11^, ^12^ the literature is scarce considering the association of both therapies in vivo. Therefore, the objective of the present study was to analyse the effects of the association of LLL (808 nm) to cell therapy with ASCs, especially on remodelling genes and on the recovery of the collagen fibres, at the 14th day of the calcaneus tendon repair.

## 2 MATERIALS AND METHODS

### 2.1 Isolation of ASC and cell culture

The procedure was done according to Yang *et  al* 13 with some modifications. Adipose tissue was obtained from the inguinal region of male Wistar (n = 2) and Lewis‐GFP (green fluorescent protein) (n = 2) rats between 90‐120 days. Adipose tissue was cut and washed in *Dulbecco's modified phosphate buffered saline *solution (DMPBS Flush, Nutricell Nutrientes Celulares, Campinas, São Paulo, Brazil) containing 2% streptomycin/penicillin. Then, 0.2% collagenase (Sigma‐Aldrich^®^ Inc., St. Louis, MO, USA) was added to ECM degradation and the solution was maintained at 37°C under gentle stirring for 1 hour to separate the stromal cells from primary adipocytes. Dissociated tissue was filtered using cell strainers (40 μm) and the inactivation of collagenase was then done by the addition of equal volume of *Dulbecco's modified Eagle's medium* (DMEM) supplemented with 15% foetal bovine serum (FBS), followed by centrifugation at 417 *g* for 10 minutes. The suspending portion containing lipid droplets was discarded and the pellet was resuspended in DMEM (containing 50 mg/L penicillin and 50 mg/L streptomycin) with 15% FBS and transferred to 75 cm^2^ bottle, maintained at 37°C with 5% CO_2_ until the 5‐6th passage (5/6P).

### 2.2 Flow cytometry

Adipose‐derived mesenchymal stem cells at 6P/7P were trypsinized and centrifuged at 1800 rpm for 10 minutes and counted using the Neubauer chamber. 1 × 10^6^ ASCs were resuspended in 200 μL of DMPBS Flush with 2% BSA (bovine serum albumin). For the immunophenotypic panel,^14^, ^15^ the following antibodies were used: CD90‐APC (eBioscience^®^), CD105‐PE (BD‐Pharmingen^TM^, San Diego, CA, USA) and CD34‐FITC double conjugated (eBioscience^®^, San Diego, CA, USA). Subsequently, ASCs were washed twice with 500 μL of DMPBS Flush and centrifuged at 2000 rpm for 7 minutes. The ASCs were resuspended in DMPBS Flush with 2% BSA, following for flow cytometry analysis.

### 2.3 In vitro differentiation potential of ASC

ASCs at 6/7P (2 × 10^4^) were plated onto 12‐wells plate and with ~80% confluency, and they were cultured using different mediums for osteogenic (n = 4) and adipogenic differentiation (n = 4). *Osteogenic differentiation*: DMEM medium supplemented with 8% FBS, 200 µmol/L of ascorbic acid, 10 mmol/L of β‐glycerophosphate and 0,5 µmol/L of dexamethasone. *Adipogenic differentiation*: DMEM medium supplemented with 15% FBS, 10 µg/mL de insulin, 100 µmol/L de indometacina (Sigma‐Aldrich^®^ Inc.) and 1 µmol/L dexamethasone. The mediums were replaced twice a week, during 30 days, and followed for the Alizarin Red‐S (0,2%) and Sudan IV (1%) staining.^16^

### 2.4 Experimental groups

A total of 90 male Wistar rats (105‐day‐old) kept at a constant temperature (23 ± 2°C) and humidity (55%) under a 12/12 hours light/dark cycle, with free access to food and water, were divided into five experimental groups: Transected (T): rats with partially transected tendons and treated with topical application of DMPBS Flush in the transected region before skin's suture; Low‐level laser (L): rats with transected tendons and treated with LLL application in the transected region after skin's suture; ASC (SC): rats with transected tendons with subsequent transplant of ASC in the transected region before skin's suture; and LLL with ASC (SCL): rats with transected tendons and treated with transplant of ASC in the transected region before skin's suture, and application of LLL after skin's suture.

### 2.5 Protocol for partial transection of the CT and application of ASCs and/or LLL

**Figure 1:**
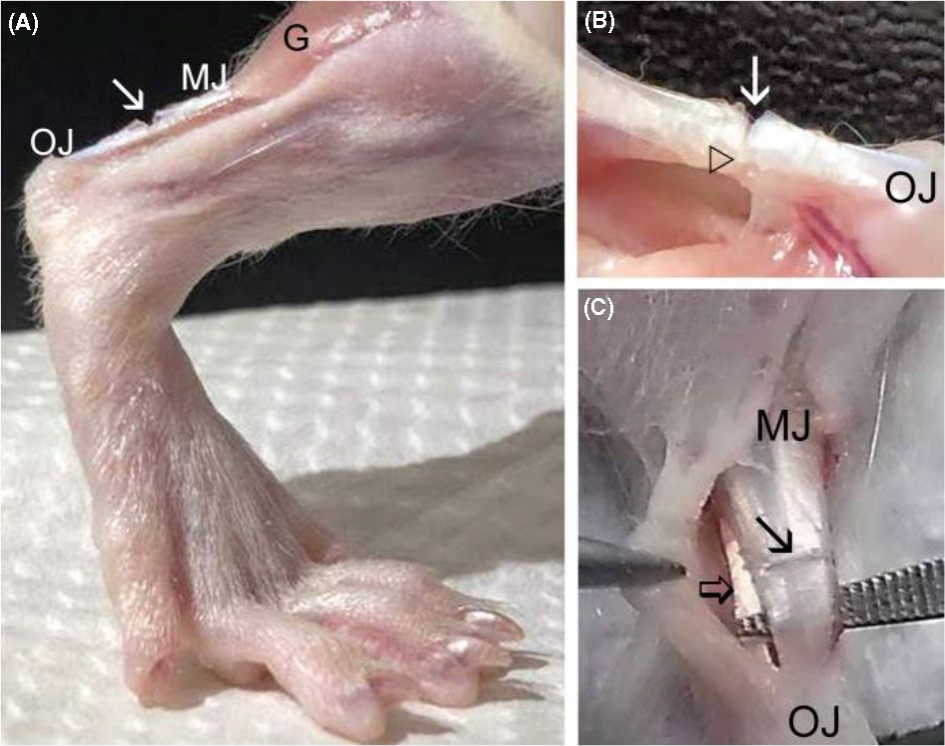
A, Model of tendon injury showing the partial transection (→) in the proximal region of the Achilles tendon of the rat hindlimb. B, Observe the partial transection (→) and the remaining portion of tendon located below the TR (△). C, Achilles tendon separated of the superficial flexor tendon (⇒) before the partial transection (→). G, gastrocnemius muscle; MJ, myotendinous junction; OJ, osteotendinous junction

The animals were anesthetized with intraperitoneal injection of Ketamine (90 mg/Kg) and Xylazine (12 mg/Kg), and the right lower paws submitted to antisepsis and trichotomy. A transverse partial transection (approximately 2/3 of the extent of the injury in depth) was performed in the proximal tendon region located at a distance of 4 mm from its insertion in the calcaneus bone (Figure 1).^17^, ^18^, ^19^ Approximately 4.5 × 10^5^ ASCs (5‐6P) were resuspended in 30 μL of DMPBS Flush and transplanted in the transected region (TR) of tendons in the SC and SCL groups, using a pipette. In tendons of T and L groups, only 30 μL of DMPBS Flush was applied. Then, the skin was sutured with nylon thread (Shalon 5‐0, Shalon Fios Cirurgicos LTDA, São Luis de Montes Belos, Goiás, Brazil) and needle (1.5 cm).

For the LLL application, the equipment used was the Model Photon Lase II® stimulator (DMC Equipamentos LTDA, São Carlos, SP, Brazil) Infrared/AsGaAl, programmed according to the Brazilian standards of medical equipment (NBR 60601‐1, NBR IEC 6061‐2‐22 and IEC 825‐1), with the parameters described in Table 1. The laser apparatus was used with pulsed light, adapted according to the study of Guerra *et al* 7 For the immobilization of the animals of L and SCL groups during the daily topical applications, a retainer rat was used and tendons received the infrared light irradiation directly on the TR performed by means of non‐contact energy delivery, within ±2 mm distance and at an angle of 90° to the surface of the injury. The treatment started 24 hours after surgery, occurring in the 1st, 3rd, 5th, 8th, 10th and 12th, totalling six applications. Euthanasia was performed on the 14th day after calcaneus tendon transection by deepening anaesthetics (Ketamine and Xylazine). All surgical and experimental protocols were approved by the Institutional Committee for Ethics in Animal Research of the Herminio Ometto Fundation‐Brazil (Protocol nº 050/2016).

**Table 1:** Parameters of LLL (Photon Lase II® stimulator [DMC] AsGaAl) used during the treatment of tendons

Model	Photon Lase II® stimulator (DMC) AsGaAl
Wave‐length	808 nm/infrared
Light	Pulsed
Power	40 mW
Beam area	0.0275 cm^2^
Intensity	50 J/cm^2^
Time	70 s
Total energy	1.3 J
Application angle	90°
Distance	2 mm
Total applications	6

### 2.6 Preparation of sections in freezing and haematoxylin‐eosin staining

Tendons (n = 3) were placed in Tissue‐Tek^®^, VWR International, Radnor, PA, USA, frozen and cut in cryostat (serial longitudinal cuts of 12 μm thickness). The sections were fixed using a 4% formaldehyde solution in Millonig buffer (0.13 mol/L sodium phosphate and 0.1 mol/L sodium hydroxide, 7.4 pH) for 20 minutes, following for haematoxylin‐eosin (HE) staining. Image analyses of the tendons were obtained using an Olympus BX53 microscope and an image analyzer (Life Science Imaging Software, Olympus Soft Imaging Solutions GmbH, Münster, Germany, Version 510_UMA_cellSens16_Han_en_00).

### 2.7 Polarization microscopy: birefringence measurements

After fixation, image analyses of the tendons (n = 3) were evaluated to detect differences in morphology based on the aggregation and organization of the collagen bundles, which reflect the variation of birefringence intensity. Birefringence properties were studied using an Olympus BX53 polarizing microscope and an image analyzer (Life Science Imaging Software, Version 510_UMA_cellSens16_ Han_en_00). Because the birefringence appears visually as brilliance, this phenomenon was measured with an image analyzer and expressed as grey average (GA) values in pixels (8 bits = 1 pixel). The larger tendon axis was positioned at 45° to the crossed analyzer and polarizer. As collagen bundles exhibit two types of birefringence: intrinsic birefringence (Bi) and form or textural birefringence (Bf).Total birefringence (the sum of Bi and Bf) was used in this study. Measurements of the TR of tendons in each experimental group were made after immersing the sections in water, a condition in which total birefringence is highly detectable. The number of measurements (120) of GA was represented as the median, and they were chosen at random in 12 sections from three tendons of each group.^20^, ^21^, ^22^

### 2.8 Immunofluorescence

For ASCs‐GFP (from Lewis rats) identification in the TR of tendons, tendon longitudinal cryosections (n = 3) were fixed in acetone (4°C) for 20 minutes. After washed in PBS (2 × 5 minutes), the sections were incubated with DAPI (0.1 mg/mL in methanol) for 5 minutes at 37°C. The sections were analysed by the fluorescence microscope (Zeiss Axio Imager.A2, Carl Zeiss AG, Oberkochen Germany) and the images captured by the Axio Vision 4.8 program. For the ASCs‐GFP quantification (fluorescence intensity) at the injured area, three sections of each animal (n = 3) of the SC and SCL groups were analysed using the Image J software (National Institutes of Health, Bethesda, MD, USA.

### 2.9 Dosage of hydroxyproline

The TR of tendons (n = 4) was cut and immersed in acetone for 48 hours, also followed by a solution containing chloroform: ethanol (2:1) for 48 hours. After dehydration, the samples were placed for drying in the oven at 37°C. Samples were weighed and hydrolysed in 6 N HCl (1 mL/10 mg of tissue) for 4 hours at 130°C according to Stegemann and Stalder,^23^ with some modifications. The hydrolysate was neutralized with 6 N NaOH, followed by spectrophotometric quantification, according to Jorge *et al* 24 The absorbance of the samples was measured at 550 nm using a microplate reader (Expert Plus; Asys^®^, Holliston, MA, USA).

### 2.10 Extraction and total proteins dosage

Samples from TR of tendons (n = 3) were frozen in liquid nitrogen and sprayed. For the extraction of total proteins, 300 μL of the T‐PER^TM^ reagent (Tissue Protein Extraction Reagent 78510; Thermo Fisher Scientific, Waltham, MA, USA) associated with the protease inhibitor (protease inhibitor cocktail ‐ 04693159001 SIGMA®) was added. The samples were then homogenized with the aid of Politron (PTA 20S model PT 10/35; Brinkmann Instruments, Westbury, NY, USA) for 40 seconds in an ice bath. The samples were then centrifuged at 12 000 rpm at 4°C for 40 minutes. The supernatant was collected for the determination of proteins by the Biureto method (Protal colorimetric method, Laborlab, São Paulo, SP, Brazil) and followed for Western blotting and ELISA analysis. Reactions were made in triplicate for each sample.

### 2.11 Western blotting

Samples (n = 3) were incubated at 100ºC for 5 minutes in 20% volume of Laemmli Buffer (0.1% bromophenol blue, 1 mol/L sodium phosphate, 50% glycerol, 10% SDS). For electrophoretic race, a volume corresponding to 50 μg of protein in biphasic gel: stacking gel (4 mmol/L EDTA, 2% SDS, 750 mmol/L base Trisma, 6.7 pH) and resolution gel (4 mmol/L EDTA, 2% SDS, 50 mmol/L base Trisma, 6.7 pH). The race was performed at 90 V for approximately 2 hours with Running Buffer (200 mmol/L base Trism, 1.52 mol/L glycine, 7.18 mmol/L EDTA and 0.4% SDS), diluted 1:4. Samples were transferred to PVDF membranes (Immun‐Blot^®^; BioRad Laboratories, Inc., Hercules, CA, USA) for 2 hours at 120 V on ice, bathed with Transfer Buffer (25 mmol/L base Trism, 192 mmol/L glycine). After transfer, the membranes were blocked with bovine albumin in basal solution for 1 hour 30 minutes at room temperature. Then, the membranes were washed three times for 10 minutes with basal solution and incubated overnight under shaking at 4°C with basal solutions plus 3% serum bovine albumin containing the following primary antibodies: caspase‐3 (ab13847, 1:500), PCNA ([PC10]ab29, 1:600), collagen I (C2456, 1:2000), collagen III (C7805, 1:4000) and β‐actin ([C4]:sc‐47778, 1:500). Subsequently, the membranes were washed three times for 10 minutes with basal solution and then incubated under agitation for 2 hours in a solution containing the following secondary antibodies: caspase‐3 (IgG‐HRP:sc‐2004, 1:10 000), PCNA, collagen I, collagen III and β‐actin (IgG1‐HRP:sc‐2060, 1:1000). Membranes were washed with basal solution and incubated for 1 minute with Thermo Scientific^®^ chemiluminescent reagents and exposed to the Syngene photodocumentator (G: BOX) for documentation. The intensity of the bands was evaluated by densitometry by the Image J program (NIH, USA), the ANOVA test and Tukey's post‐test (*P* < 0.05) were performed in GraphPadPrism® software version 3.0 (GraphPad Software, San Diego, CA, USA).

### 2.12 Enzyme‐Linked Immunosorbent Assay (ELISA)

The concentration of pro‐ (tumour necrosis factor, TNF‐α) and anti‐(Interleukin‐10, IL‐10) inflammatory cytokines was analysed by ELISA using monoclonal antibodies to each murine cytokine Pharmingen (BD‐Pharmingen™, San Diego, CA, USA) or RD Systems (R&D Systems, Inc., Minneapolis, MN, USA). A volume corresponding to 30 μg of proteins from each sample (n = 3) was added to the immunoassay plate for determination of TNF‐α and IL‐10 levels separately, according to manufacturer's instructions (Pharmingen, BD). Reactions were made in triplicate for each sample. The absorbance was measured at 450 nm on a microplate reader, and the concentrations of each cytokine were determined on the basis of the linear regression line made for the standard curve obtained as the appropriate recombinant cytokine standard.

### 2.13 Real‐time PCR Array

The TR of tendons was collected (n = 3), placed in stabilizing solution (RNA‐later; QIAGEN^®^, Hilden, Germany) and maintained at −20°C. Total RNA extraction was made according to Marqueti *et al* 25 and using the RNeasy® Fibrous Tissue Mini Kit (QIAGEN^®^), following the manufacturer's instructions. 120 ng of RNA of each sample was used for the synthesis of cDNA, using the *RT^2^* *First Strand Kit* (QIAGEN^®^) and thermocycler Mastercycler Pro (Eppendorf^®^, Hamburg, Germany), also following the manufacturer's instructions. The RT‐PCR array reaction was performed using the *RT^2^* *Profiler PCR Arrays* (A format) kit in combination with the *RT^2^ SYBR Green Mastermixes* (QIAGEN^®^) on the thermocycler apparatus Stratagene MX300SP (Thermo Fisher Scientific), following the manufacturer's instructions. Three types of reaction controls were used for each animal sample: (a) Positive PCR control; (b) Reverse transcriptase control; and (c) Control for contamination of rat genomic DNA. The *Glyceraldehyde‐3‐phosphate dehydrogenase* (*Gapdh*, NM_017008) was used as endogenous control for each sample. The following genes were analysed (QIAGEN^®^): *Scleraxis* (*Scx*, NM_001130508); *Tenomodulin *(*Tnmd*, NM_022290); *Interleukin 1 beta* (*II1b*, NM_031512); *Transforming growth factor, beta 1* (*Tgfb1*, NM_021578); *Matrix metallopeptidase 2* (*Mmp2*, NM_031054); *Matrix metallopeptidase 9* (*Mmp9*, NM_031055); *TIMP* *metallopeptidase inhibitor 2* (*Timp2*, NM_021989); *Decorin* (*Dcn*, NM_024129); *Lysyl oxidase* (*Lox*, NM_017061); and e *Growth differentiation factor 5 *(*Gdf5*, XM_001066344). Reactions were made in a single cDNA pipetting for each gene including endogenous control. T group was used as calibrator sample, and for each target gene, the 2^−∆∆^ *^C^* ^T^ method was used to calculate the relative expression level (fold change). The results were represented as the relative gene expression in comparison with the calibrator sample that is equal to 1.

### 2.14 Statistical analysis

For biochemical and molecular analyses, data from different experimental groups were analysed by the analysis of variance (ANOVA), followed by the Tukey test (*P* < 0.05). The Mann‐Whitney *U* test (*P* < 0.05) was used only for analysis of the birefringence measurements, also using the GraphPad Prism^®^ (GraphPad Software, La Jolla, CA, USA), version 3.0. All results were presented as the mean and standard deviation.

## 3 RESULTS

### 3.1 In vitro adipogenic and chondrogenic differentiation of ASCs and positive ASCs markers

**Figure 2:**
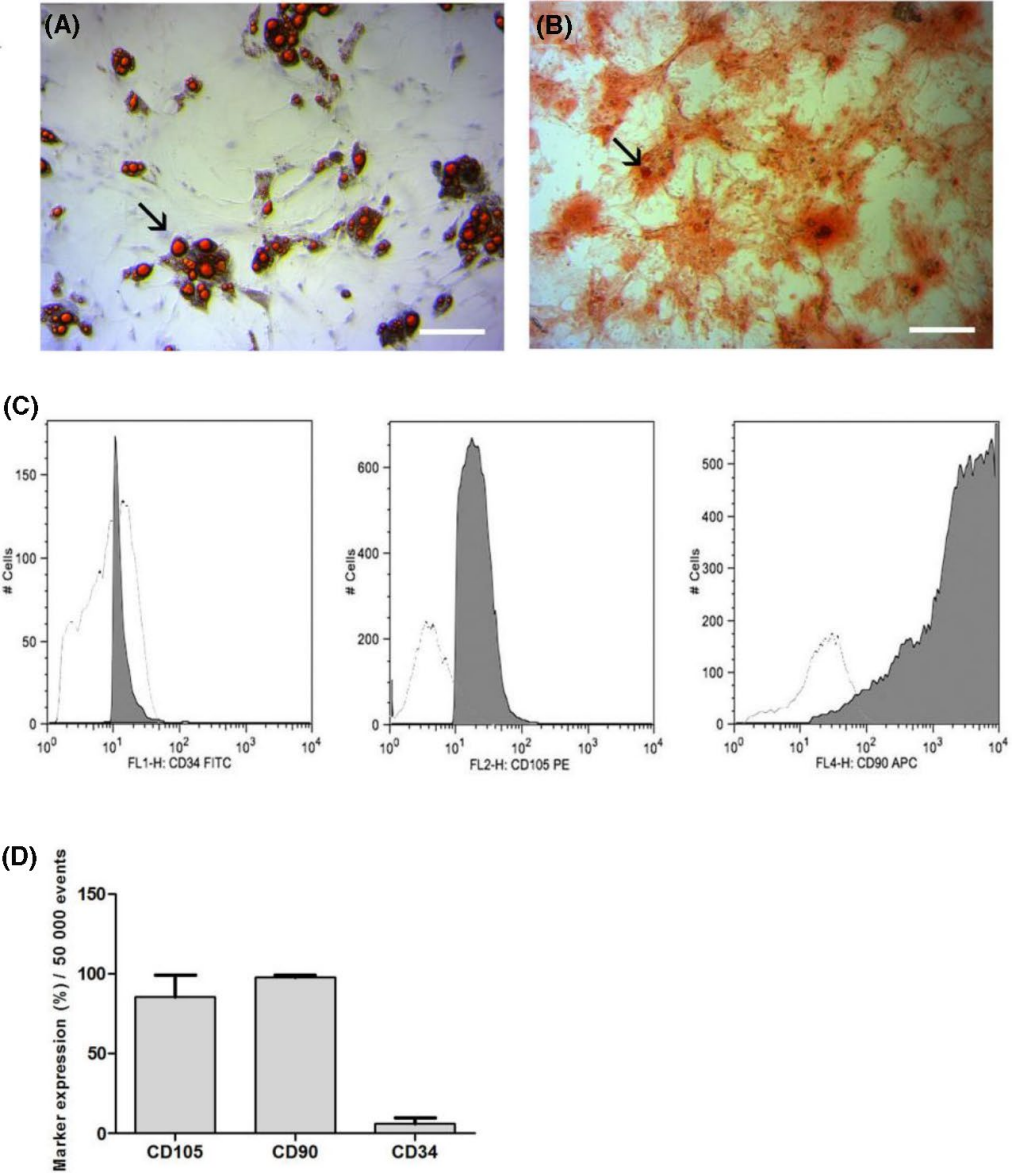
A, Adipogenic differentiation evidenced by Sudan IV staining and counterstaining with hematoxylin: observe red‐intracellular lipid droplets (→). B, Osteogenic differentiation after staining with Alizarin Red SD: observe red stained calcification spots in ECM (→). C, Histograms demonstrate the *x*‐axis fluorescence scale considered positive when the cell peak is above 101 (CD34 and CD 105) or 102 (CD90), and controls for ‐APC,‐PE and ‐FITC (D), corresponding to non‐marked cells due very low fluorescence. E, Flow cytometry for characterization of ASCs: the graph shows high expression of CD105 markers (85.30% ± 23.90%) and CD90 (97.50% ± 3.12%), and low expression of CD34 (6.27% ± 5.73%).Bars = A, B, D: 120 μm; C: 40 μm

ASCs (6‐7P) cultures stained with Sudan IV and Alizarin Red SD exhibited red‐intracellular lipid droplets and calcification spots, respectively (Figure 2A,B). Flow cytometry showed high expression of CD105 markers and CD90, and low expression of CD34 (Figure 2C‐E).

### 3.2 Cell migration assay

**Figure 3:**
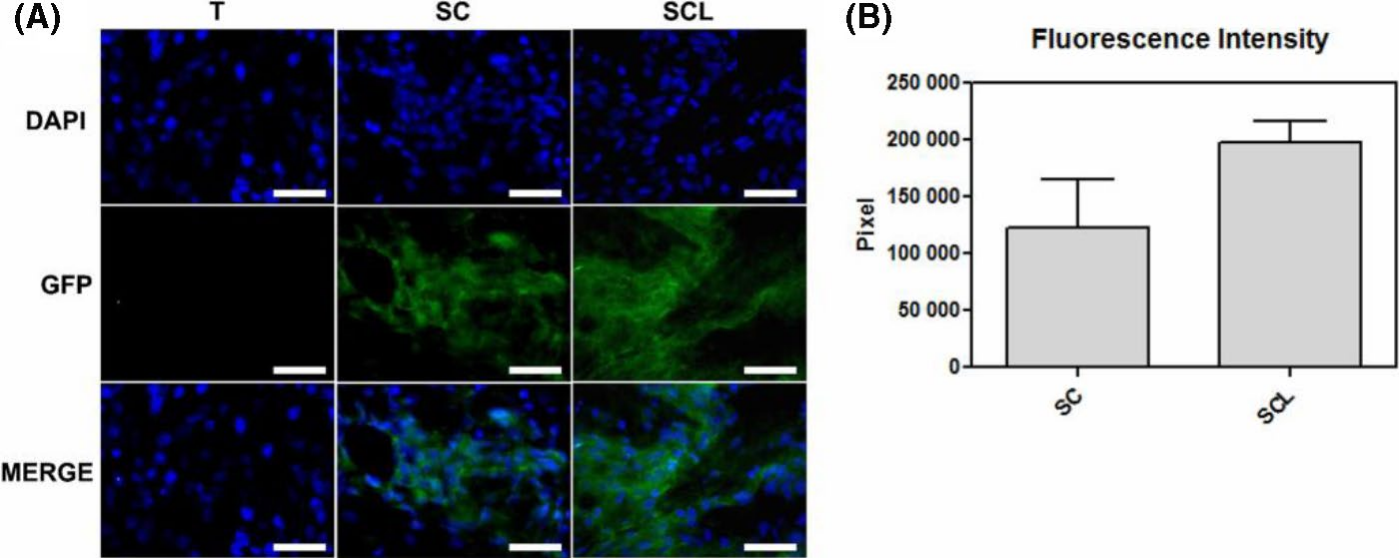
A, Cell migration analysis: observe the nuclei labeled with DAPI (blue) in all groups and the presence of ASCs‐GFP, represented by the green cytoplasm only in the SC and SCL groups on the 14th day after the injury. B, Fluorescence intensity of ASCs‐GFP at the injured area of tendons. Bars = 20 μm

On day 14, GFP‐ASCs were quantified in the TR of tendons. It was observed a tendency to increase of the ASCs in tendons of SCL group in comparison with SC (Figure 3).

### 3.3 Genes expression analysis

**Figure 4:**
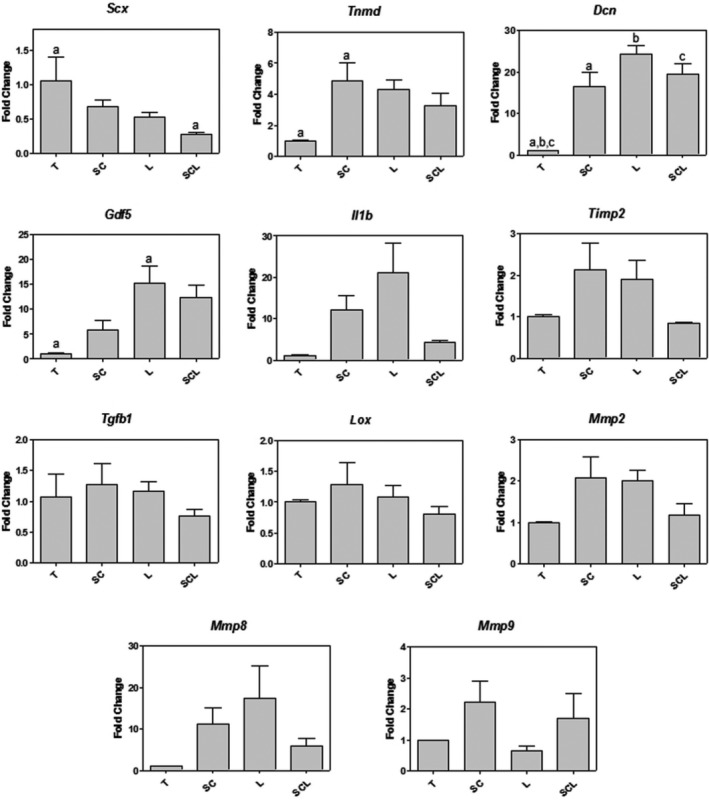
RT‐PCR array for gene expression analysis, with differences for the Scx, Tnmd, Dcn and Gdf5 genes. Significant differences between groups marked with the same letter (*P* < 0.05)

There was lower expression of *Scleraxis* (*Scx*) in the SCL group, higher expression of *Tenomodulin* (*Tnmd*) in the SC group and of *Growth differentiation factor 5 (Gdf5)* in the L group, compared to the T group. The expression of *Decorin* (*Dcn*) was increased in all treated groups, SC, SCL and L respectively, compared to the T group (Figure 4).

### 3.4 Pro‐ and anti‐inflammatory cytokines quantification

**Figure 5:**
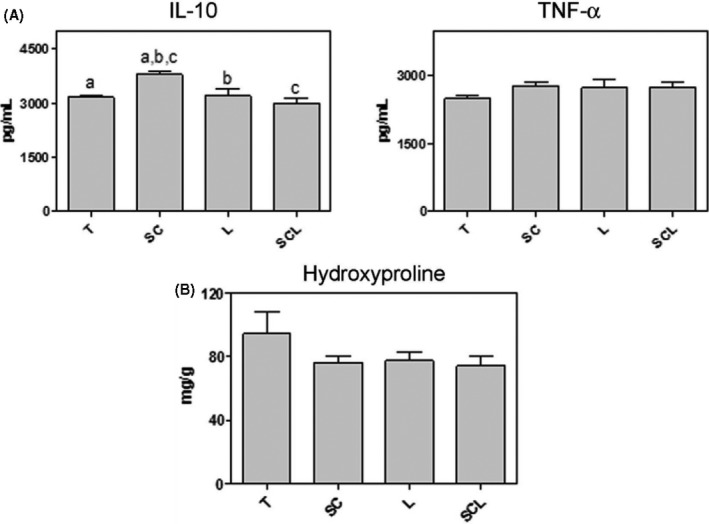
A, ELISA for the analysis of cytokines IL‐10 and TNF‐α (pg/mL). Significant differences between groups marked with the same letter (*P* < 0.05). B, Hydroxyproline concentration (mg/g tissue) of tendon RT: there was no significant difference between the experimental groups

The graph for TNF‐α and IL‐10 demonstrated a significant increase in IL‐10 concentration in the SC group compared to all other groups. Regarding TNF‐α, there were no significant differences between the groups (Figure 5A).

### 3.5 Total collagen quantification

No significant difference was observed in the quantification of hydroxyproline between the analysed groups (Figure 5B).

### 3.6 Collagen I and III, PCNA and Tnmd quantification

**Figure 6:**
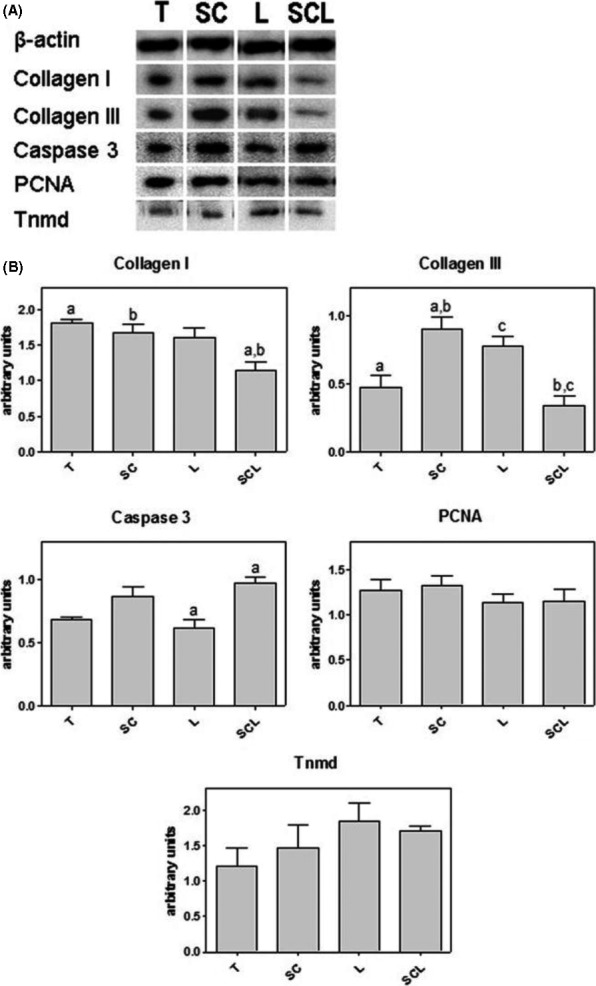
A, Western blotting for collagen I and III, caspase‐3, PCNA and Tnmd. Endogenous control: β‐actin. B, Densitometry of the WB bands for each protein. Significant differences between groups marked with the same letter (*P* < 0.05)

The amount of collagen I was lower in the SCL than in the T and SC groups. For collagen III, the SC group had a higher amount when compared to T and SCL. The L group showed a significant increase compared to only the SCL group. The amount of caspase‐3 was decreased in the L group in relation to SCL. No differences were observed in the amounts of PCNA (Proliferating Cell Nuclear Antigen) and Tnmd between the different groups (Figure 6).

### 3.7 Collagen fibres organization measurement

**Figure 7:**
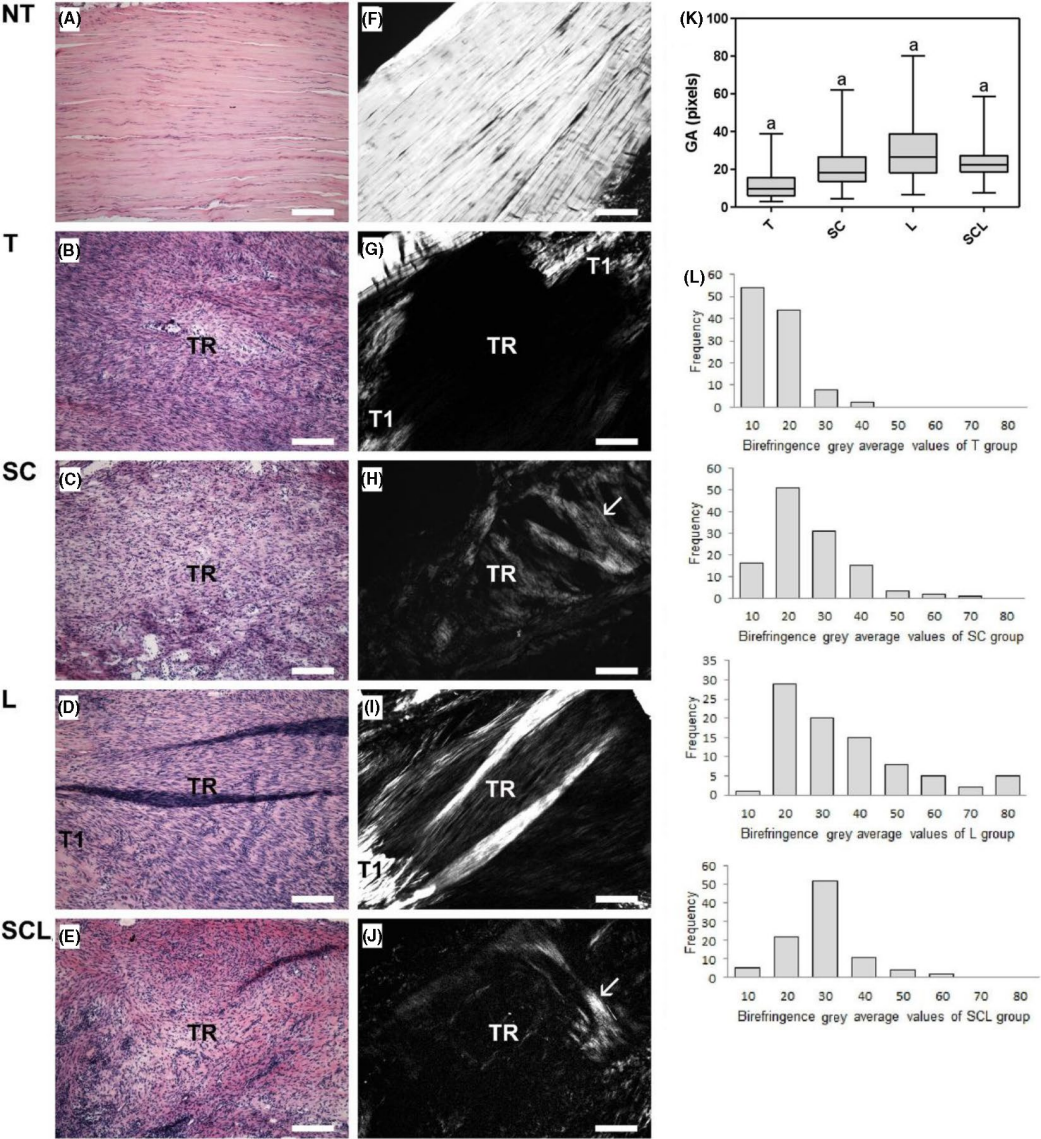
A‐E, Longitudinal section of the transection region (TR) of TC stained with HE: observe low cellularity in normal tendon (A) and increased cellularity in TR (B‐E). F,J, Images analysed under polarization microscopy (without staining), where the largest axis of the tendon was positioned at 45º between the polarizers: observe intense brightness of the collagen fibers in the normal tendon (F) due to the high degree of organization of the collagen bundles. Crimp: undulation pattern of the collagen fibers evidenced by light and dark regions. In the tendons of the transected groups, observe low birefringence in the TR due to the disorganization of the fibers (G‐J). Observe T1, located in the adjacency of TR, exhibiting higher brightness. In the groups SC (H) and SCL (J) observe the presence of fibers perpendicular to the greater axis of the tendon, located in the TR indicated by the arrows. In the L (I) group, brighter regions are observed. K, Birefringence gray average (GA) measures: Significant differences between groups marked with the same letter (*P* < 0.05). L, Histogram showing the distribution frequency of the birefringence values of the different groups. Bars: 200 μm

Images of tendons analysed under polarization microscopy showed differences in the collagen organization in the TR of all groups (Figure 7). The birefringence measurements (Figure 7K) showed higher values in the L group, followed by SCL, SC and T groups. In the images of HE‐stained tendons (Figure 7A‐E), the general organization of TR was observed, with higher cellularity in the transected groups compared to the N group (Figure 7D).

## 4 DISCUSSION

In the present study, the cell migration assay showed the presence of ASCs‐GFP in the TR on the 14th day after their application to the tendon, proving the incorporation of the ASCs into the tissue during the repair process. A tendency towards higher fluorescence intensity of the ASCs‐GFP was observed in the SCL group compared to the SC. In general, a small percentage of ASCs survives at the injury site after application.^9^ A previous study of our group has shown the presence of transplanted ASCs on 3rd and 14th days after tendon transection, probably in response to the intense initial inflammatory process.^19^

During the tendon repair, TGF‐β1,^26^ Il‐10^27^ and scleraxis^28^, ^29^ are responsible for the stimulation of collagen synthesis. No differences were observed in the expression of *Tgfb1* among groups, but the higher concentration of IL‐10 in the SC group corroborates the higher amount of collagen III and I in this group, compared to the T and SCL groups, respectively. Data of Behrendt *et  al* 27 showed the involvement of IL‐10 in the formation of ECM through the stimulation of collagen production during the proliferative phase of cartilage repair. Collagen III forms a temporary support matrix that assists in the deposition of new collagen I fibres, and subsequent formation of collagen bundles in the next stages of the tissue repair process.^27^ Shen *et  al* 30 also observed increased collagen I and III in a co‐culture system of corneal stromal cells and ASCs, corroborating with our data.

During the calcaneus tendon repair using LLL, a study of Abid and Abid^31^ demonstrated a higher collagen deposition and significant number of tenocytes. LLL therapy did not show the same results in our study, since no difference was observed in the amount of total collagen and collagen types I and III, as well as in the amount of Tnmd, a marker of tenocytes. The lower amount of collagen I in the SCL group in relation to the T group may be related to the lower expression of *Scx* in SCL *Scx* is a transcription factor that directly regulates the gene expression of collagen I in tendons.^29^ *Scx* is also a key regulator of tenocyte differentiation, being detected in populations of tendon precursor cells.^3^, ^32^ However, our results did not demonstrate a relationship between higher expression of *Scx* and *Tnmd*, which would indicate the performance of *Scx* in the differentiation of ASCs into tenocytes. *Tnmd* is a marker of differentiated fibroblasts^32^ and acts on the self‐renewal of tendon progenitor cells, preventing tissue senescence and exerting a regulatory role on cell proliferation.^32^ No difference was observed in the amounts of PCNA, a marker of cell proliferation, although some studies demonstrate the performance of ASCs in increased cell proliferation in tendon repair.^8^, ^12^, ^33^ A study of Dex *et al* 34 also demonstrates the role of *Tnmd* in the maturation of collagen fibres. Therefore, although no significant difference was detected in the protein quantification of Tnmd, there was a trend towards a higher amount of Tnmd in the L, SCL, SC and T groups, respectively, corroborating the higher degree of organization of the collagen fibres.

The SCL group presented higher amount of caspase‐3 in relation to the L group, suggesting an increase in apoptosis. Studies show the positive effects of ASCs^8^, ^12^, ^33^ and of LLL^35^ in increased cell proliferation in tendon repair and during others tissue regeneration.^36^ Our data showed no difference in the proliferator marker, as well showed increased apoptosis after association of treatments in comparison with LLL. It is important to emphasize that the increased caspase‐3 amount may be due to the apoptosis of inflammatory cells present in small quantities at this stage of tendon repair. Burgon and Megeney^37^ demonstrated that caspase‐3 in myoblasts may present a cell signalling pathway capable of managing cell differentiation without causing apoptosis. Due to the absence of differences between the amounts of Tnmd among groups, it was not possible to correlate caspase‐3 as inducer of cell differentiation in the SCL group.

Our results do not demonstrate differences in the amount of TNF‐α in the different groups, although the literature describes the anti‐inflammatory role of LLL^38^ and ASCs.^39^ Regarding the expression of *Il1b*, another proinflammatory cytokine,^40^ it was observed a marked tendency to increase in the SC and L groups, corroborating with tendency to increase of *Mmp8 *gene expression in the same groups. *Il1b* has been identified as potent inducers of matrix metalloproteinases (MMPs).^41^

The L group presented greater expression of *Gdf5*, a gene involved in several pathways, including the reorganization of the ECM under tissue repair,^42^ corroborating with the larger organization of the collagen fibres observed in this group. Chhabra *et al* 42 used knockout mice for the *Gdf5* gene and observed calcaneus tendon repair delay, since the mutant animals presented less proteoglycans and collagen, as well as less organization of the collagen fibres. Also corroborating the greater organization of the collagen fibres after the different treatments, the expression of *Dcn* was significantly higher in all treated groups compared to the T group. *Dcn* maintains the structure of the collagen fibrils by regulating the diameter and realignment of the fibres, as well as the mechanical properties of the tendons.^43^ No significant differences were observed among groups according to data related to the expression of *Mmp2*, *Mmp9*, *Timp2* and *Lox*, genes responsible for the control of ECM degradation and remodelling. However, the literature demonstrates the effect of LLL and ASCs on these enzymes in a protein level.^7^, ^30^, ^44^

Low‐level laser‐treated group presented a larger organization of the fibres, followed by the SCL, SC and T groups. Studies using LLL or ASCs in tendon repair also demonstrated their role in the reorganization of the collagen matrix^6^, ^7^, ^19^ corroborating with our results. Possibly, the association of treatments was less effective because both therapies present anti‐inflammatory effects.^7^, ^8^, ^39^ After lesion, the release of several growth factors and cytokines during the inflammation process is responsible for activation of genes responsible for the matrix remodelling.^17^, ^19^, ^41^, ^45^ This hypothesis, although speculative, can be considered a limitation of the present study, in which no signalling pathways related to inflammation were analysed at the beginning of the repair process.

According to our data, transplanted ASCs migrated to the transected region and increased collagen III and IL‐10. After LLL treatment, the higher amount of collagen III was possibly related to an increased *Gdf5* gene expression. The LLL and ASCs in combination down‐regulated the expression of *Scx* gene, diminished collagen I and III, and increased caspase‐3. In conclusion, all treatments differently altered the expression of important remodelling genes, especially of *Dcn*. The LLL isolated was the most effective in recovering of the collagen fibres organization, and when combined with the ASCs, the LLL improved the cells performance in the recovery of the collagen fibres. Some limitations of this work are the absence of biomechanical parameters and of analysis of the gait, which will be considered in a next study, together with other experiments to elucidate the cellular signalling involved in the combination of these therapies during the tendon repair.

## AUTHOR CONTRIBUTIONS

The authors Andrea A. de Aro and Edson R. Pimentel had the same contribution during the supervision of this work.
